# Efficacy of Dabrafenib Plus Trametinib Combination in Patients with *BRAF* V600E-Mutant NSCLC in Real-World Setting: GFPC 01-2019

**DOI:** 10.3390/cancers12123608

**Published:** 2020-12-02

**Authors:** Jean-Bernard Auliac, Sophie Bayle, Pascal Do, Gwenaëlle Le Garff, Magali Roa, Lionel Falchero, Eric Huchot, Gilles Quéré, Gaëlle Jeannin, Anne-Cécile Métivier, Joëlle Hobeika, Florian Guisier, Christos Chouaid

**Affiliations:** 1 Department of Pneumology, CHI de Créteil, 94000 Créteil, France; joelle.hobeika@ght-novo.fr (J.H.); Christos.Chouaid@chicreteil.fr (C.C.); 2 Department of Pneumology, CHU de Saint-Etienne, 42055 Saint-Etienne, France; sophie.bayle@chu-st-etienne.fr; 3 Department of Pneumology, Centre Baclesse, 14076 Caen, France; pdo@baclesse.unicancer.fr; 4 Department of Pneumology, CH de Saint Brieuc, 22027 Saint-Brieuc, France; gwenaelle.legarff@ch-stbrieuc.fr; 5 Department of Pneumology, CHI de de Fréjus Saint Raphael, 83600 Fréjus, France; roa-m@chi-fsr.fr; 6 Department of Pneumology, Hôpital Nord Ouest de Villefranche-sur-Saône, 69400 Gleizé, France; lfalchero@lhopitalnordouest.fr; 7 Department of Pneumology, CHU Réunion Site Sud, 97448 Saint-Pierre, France; eric.huchot@chu-reunion.fr; 8 Department of Pneumology, CHU de Brest, 29200 Brest, France; gilles.quere@chu-brest.fr; 9 Department of Pneumology, CHU de Clermont-Ferrand, 63000 Clermont-Ferrand, France; gjeannin@chu-clermontferrand.fr; 10 Department of Pneumology, Hôpital Foch, 92150 Suresnes, France; ac.metivier@hopital-foch.com; 11 Department of Pneumology, CHU Charles Nicolle, 76000 Rouen, France; florian.guisier@chu-rouen.fr

**Keywords:** *BRAF* mutations, dabrafenib, non-small-cell lung cancer, oncogenic driver, targeted therapy, trametinib, V600E mutation

## Abstract

**Simple Summary:**

Mutations of the *BRAF* oncogene are reported in tumors of patients with non-small-cell lung cancer in 2–4% of cases (about half of them are V600E mutation). Dabrafenib plus trametinib combination is approved in Europe for *BRAF* V600E-mutant metastatic non-small-cell lung cancer. However, there are few published data on the efficacy and safety of this combination outside of formal clinical trials. In this retrospective multicentric observational study, we describe in a real-life setting the characteristics and the outcomes of patients with *BRAF* V600E-mutant NSCLC treated with the dabrafenib and trametinib combination. The results in 40 patients suggest that efficacy and safety of dabrafenib plus trametinib combination in patients with metastatic non-small-cell lung cancer harboring this mutation are comparable in a real-world setting and in clinical trials for both previously untreated and treated patients.

**Abstract:**

Dabrafenib plus trametinib combination is approved in Europe for *BRAF* V600E-mutant metastatic non-small-cell lung cancer (NSCLC). The objective of this study was to assess efficacy and safety of this combination in a real-world setting. This retrospective multicentric study included 40 patients with advanced NSCLC harboring *BRAF* V600E mutation and receiving dabrafenib plus trametinib. The median progression-free survival (PFS) and overall survival (OS) were 17.5 (95% CI 7.1–23.0) months and 25.5 (95% CI 16.6–not reached) months in the entire cohort, respectively. For the 9 patients with first-line treatment, median PFS was 16.8 (95% CI 6.1–23.2) months and median OS was 21.8 (95% CI 1.0–not reached) months; for the 31 patients with second-line or more treatments, median PFS and OS were 16.8 (95% CI 6.1–23.2) months and 25.5 (95% CI 16.6–not reached) months, respectively. Adverse events led to permanent discontinuation in 7 (18%) patients, treatment interruption in 8 (20%) and dose reduction in 12 (30%). In conclusion, these results suggest that efficacy and safety of dabrafenib plus trametinib combination in patients with *BRAF* V600E metastatic NSCLC are comparable in a real-world setting and in clinical trials for both previously untreated and treated patients.

## 1. Introduction

Mutations of the *BRAF* oncogene are reported in tumors of patients with non-small-cell lung cancer (NSCLC) in 2–4% of cases [1]. In about half of cases, the mutation is due to the substitution of glutamate to valine at codon 600 in the exon 15 of the *BRAF* kinase domain (V600E mutation). This mutation strongly activates the RAF-ERK signaling pathway that acts as an oncogenic driver by promoting cell growth and proliferation and inhibiting apoptosis [2].

The success of BRAF inhibitors in melanoma led to similar studies in NSCLC. It was shown in both melanoma and NSCLC that the concomitant BRAF inhibition with dabrafenib and inhibition of the downstream mitogen-activated protein kinase (MEK) with trametinib improved clinical response compared to BRAF inhibition alone [1]. A phase II trial showed that dabrafenib in combination with trametinib could be considered as a new therapy with significant antitumor activity and manageable safety profile in previously treated NSCLC patients with BRAF V600E mutation [3]. This 14-month-long international study included 59 patients from 30 centers across North America, Europe and Asia; of those patients, 36 (63.2%; 95% CI 49.3–75.6) achieved an investigator-assessed overall response. Comparable results were reported in a population of 36 untreated patients, with 23 (64%, 95% CI 46–79) achieving an overall response rate, 2 (6%) patients achieving a complete response and 21 (58%) achieving a partial response [4]. The data were updated at the last ASCO conference in 2020 [5]. In cohorts of untreated and previously treated patients, the ORR was 63.9% (95% CI 46.2–79.2) and 68.4% (95% CI 54.8–80.1), and median PFS was 10.8 (95% CI 7.0–14.5) months and 10.2 (95% CI 6.9–16.7) months. Median OS was 17.3 months (95% CI 12.3–40.2; 3-year OS: 40%) and 18.2 months (95% CI 14.3–28.6; 3-year OS: 33%) with 14/36 and 11/57 patients alive in treatment-naive and pretreated patients, respectively.

Apart from this combination, only encorafenib, a highly selective BRAF inhibitor, is currently being studied in this population (NCT04526782). The European Medicines Agency and the Food and Drug Administration approved the dabrafenib and trametinib combination for patients with stage IV NSCLC harboring *BRAF* V600E mutation. 

There are few published data on the efficacy and safety of dabrafenib and trametinib combination outside of formal clinical trials. The objective of this retrospective multicentric observational study was to describe in a real-life setting the characteristics and the outcomes of patients with *BRAF* V600E-mutant NSCLC treated with dabrafenib and trametinib combination.

## 2. Results

A total of 40 patients treated with dabrafenib plus trametinib from October 2015 to March 2019 were included in 14 centers. Patients had a mean age of 70.1 years (from 49 to 94 years), and 55% of patients were women (Table 1). All patients had *BRAF* V600E mutation without concomitant mutation and almost all had adenocarcinoma (95%). Of the patients studied, 67.5% were former or current smokers. ECOG performance status was 0–1 at diagnosis for 87% of patients, and 76% had a weight loss >5%. Stage IV was achieved at diagnosis for 75% of patients. 

At initiation of dabrafenib plus trametinib, stage IV was achieved for 95% of patients (72% of patients with two or more metastatic sites) (Table 1). The main metastatic sites were pleura (48%), bone (40%), lung (33%) and brain (13%).

Nine patients received dabrafenib plus trametinib combination as first line and 31 patients as second line and more (second line, *n* = 15; third line, *n* = 8; fourth line and more, *n* = 8). For the 31 patients with prior treatments before dabrafenib plus trametinib platinum-based doublet, chemotherapy was the main (90%) first-line treatment. 

At the cut-off date for analysis (October 2020), after a median follow-up of 16.5 months (range, 0.1–29.3 months), 19 patients were alive and 29 progressed under dabrafenib plus trametinib treatment. Eight patients continued dabrafenib plus trametinib treatment after progression. 

Median treatment duration with dabrafenib plus trametinib was 14.1 months for all patients (17.5 months for the 9 patients with dabrafenib plus trametinib as first line and 14.1 months for the 31 patients with dabrafenib plus trametinib as second line). 

**Figure 1:**
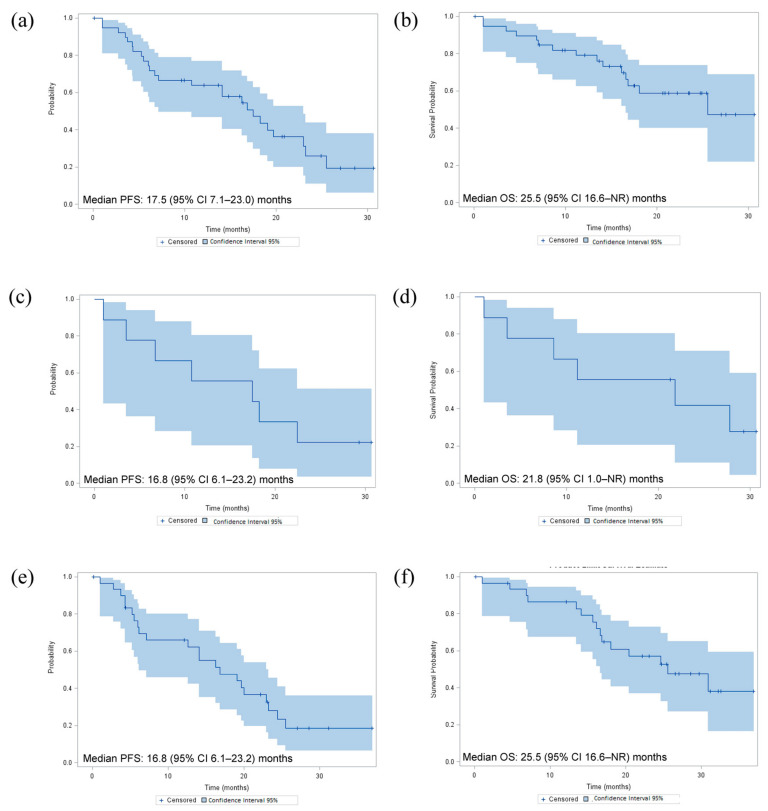
Progression-free survival (PFS) and overall survival (OS) in all patients (*n* = 40) treated with dabrafenib plus trametinib combination (**a**,**b**, respectively), in the subgroup of patients (*n* = 9) who received dabrafenib plus trametinib as first-line treatment (**c**,**d**) and in the subgroup of patients (*n* = 31) who received dabrafenib plus trametinib as second line or more (**e**,**f**). NR, not reached.

Median PFS and OS were 17.5 (95% CI 7.1–23.0) months and 25.5 (95% CI 16.6–not reached) months in all patients, respectively (Figure 1). Comparable survival values were obtained for the subgroups according to the line of treatment with dabrafenib plus trametinib (Figure 1).

Adverse events led to permanent discontinuation in 7 (18%) patients (heart failure, 2 patients; kidney failure, 1; cytolytic hepatitis, 1; deterioration of the general condition with arthralgia, 1; unknown, 2), treatment interruption in 8 (20%) and dose reduction in 12 (30%). 

## 3. Discussion

The present study included patients with *BRAF* V600E-mutant advanced NSCLC. These patients are known to have a different profile than those with other genomic alterations (EGFR, ALK, ROS, HER2, etc.) Our cohort appears to be representative of patients with *BRAF* V600E-mutant advanced NSCLC with 71% smokers despite a slightly older age (70.1 years) and a slight majority of women (55%). Paik et al. [6] reported that NSCLC patients with *BRAF* mutations are typically current or former smokers in contrast to patients with *EGFR* mutations and *ALK* rearrangements who are mostly never smokers. These characteristics are also comparable with the data of other studies. In the 262 patients with *BRAF* mutation reported by Barlesi et al. [7], the characteristics were as follows: age, 65.9 years; women, 39%; smokers, 75%; adenocarcinoma, 87%. For the 57 and 36 patients of the two pivotal studies that supported the marketing authorization of the dabrafenib plus trametinib combination, the characteristics were as follows: age, 64 and 67 years; women, 49% and 31%; smokers, 72% and 72%; adenocarcinoma, 98% and 89%, respectively [3,4]. 

In the present cohort, the median PFS was 17.5 (95% CI, 7.1–23.0) months and the median OS was 25.5 (95% CI 16.6–not reached) months. Despite the absence of comparative studies, available data suggest that the efficacy of dabrafenib plus trametinib is superior to chemotherapy. Thus, in 46 advanced NSCLC patients enrolled in 2012–2014 in France, we previously reported that carriers of the V600E mutation had a PFS of 8.2 (95% CI 5.9–19.0) months: 8.7 (95% CI 6.4–15.2) months for first-line therapy and 4.1 (95% CI 2–10.9) months for second-line treatments [8]. Patients treated with BRAF-targeted therapy (vemurafenib) had a PFS of 9.2 (95% CI 6.4–22.2) months. The efficacy data reported in this real-life study with unselected patients were close to outcomes from the two phase II studies which demonstrated the clinical efficacy of dabrafenib plus trametinib in patients with stage IV *BRAF* V600E-mutant NSCLC. Thus, in cohort B (previously treated patients), PFS and OS were 10.2 (95% CI 6.9–16.7) months and 18.2 (95% CI 14.3–28.6) months, respectively; in cohort C (previously untreated patients), PFS and OS were 10.8 (7.0–14.5) months and 17.3 (12.3–40.2) months, respectively [3,4,5,9]. PFS and OS appeared to be slightly improved in previously untreated patients than in previously treated patients. Due to the small number of previously untreated patients in our cohort, we cannot confirm this improvement. 

These studies also suggest that BRAF dual therapy is superior to BRAF monotherapy. Thus, in the retrospective EURAF study, 35 patients with advanced NSCLC harboring *BRAF* mutations received different BRAF inhibitors (vemurafenib, dabrafenib or sorafenib). Overall, median PFS was 5 months and median OS was 10.8 months [10]. More recently, immunotherapy became an important tool for the treatment of NSCLC. According to the literature, anti-PD-L1 agents are similarly active in the BRAF-mutated tumors and their undruggable counterparts. In a recent study of our group in 26 *BRAF* V600E patients treated with anti-PD1/PDL-1 as second line or more, median PFS and OS were 5.3 (95% CI 2.1–not reached) months and 22.5 (95% CI 8.3–not reached) months, respectively. Although these results obtained in a small number of patients should be considered cautiously, they suggest that immunotherapy could be an option in patients with progression under targeted therapy [11]. 

In this real-life study, the safety profile was acceptable and adverse events were manageable, leading to permanent discontinuation in 7 (18%) patients, treatment interruption in 8 (20%) and dose reduction in 12 (30%). These results are comparable to data from the Phase II studies of Planchard et al. [3,4] where adverse events led to permanent discontinuation, treatment interruption and dose reduction in 12%, 61% and 35% of previously treated patients and in 22%, 75% and 39% of untreated patients, respectively.

The study has the limitations of a retrospective study with possible unknown selection biases. Another limitation is the definition of disease progression by physicians, as usual. Due to the rarity of the *BRAF* V600E mutation, setting up clinical studies and including a cohort of patients with sufficient sample size within a reasonable time is a challenge. Only nine patients received dabrafenib plus trametinib as a first-line treatment. Therefore, efficacy results obtained in this subgroup must be considered cautiously.

## 4. Materials and Methods 

This retrospective observational multicenter study was performed in French centers. The objective was to describe in a real-world setting the clinical characteristics and the outcomes of patients with advanced *BRAF* V600E-mutant NSCLC treated with dabrafenib plus trametinib combination. 

The primary objective of the study was to evaluate progression-free survival (PFS) and overall survival (OS) from dabrafenib plus trametinib initiation. The secondary objectives were to describe patient characteristics, PFS and OS according to subgroups (dabrafenib plus trametinib as a first-line treatment or second-line treatment or more) and safety of dabrafenib plus trametinib. 

The study was conducted in accordance with the Declaration of Helsinki. Participating centers were responsible for obtaining patient consent and institutional approval (“*Comité Indépendant de Traitement des Ressources de Santé*”, CITRS, authorization 914,146, valid for all centers).

All contributors were trained in good clinical practices. The study was purely an academic collaboration and was not funded by industry 

Centers were asked to include all their consecutive adult patients with *BRAF* V600E-mutant NSCLC treated with dabrafenib and trametinib regardless of treatment lines. According to the Summary of Product Characteristics, patients received dabrafenib (150 mg BD per os) plus trametinib (2 mg OD per os) either as first-line treatment or as second-line or more treatment. Molecular analysis of *BRAF* is performed in France routinely in a network of regional molecular genetics centers. This systematic testing has been funded by the Ministry of Health for all patients with advanced nonsquamous NSCLC and also for nonsmoking patients with squamous NSCLC. 

Patient demographics and clinical characteristics at NSCLC diagnosis including age, sex, smoking status, tumor histology, cancer stage, number and sites of metastases, ECOG performance status, loss of weight and cancer-related symptoms were collected from patient files. Information on lines of treatments received before dabrafenib plus trametinib was collected from diagnosis and included treatment sequences and type of therapy. Clinical outcomes included date of death and dates of any clinician-defined progression based on increased lesion size, appearance of new lesions or symptomatic findings. Missing data were not replaced.

PFS was defined as the time from dabrafenib plus trametinib initiation to progression on dabrafenib plus trametinib. Progression was defined as Response Evaluation Criteria in Solid Tumors version 1.1 criteria (RECIST 1.1) radiological or clinical progression (deteriorated clinical status preventing systemic treatment) or death. Assessments were done in each participating center without centralized imaging review. OS was calculated from the start of dabrafenib plus trametinib treatment to death. The Kaplan–Meier method was used to estimate PFS and OS for the entire cohort and according to treatment lines; the 95% CIs were calculated using the Greenwood formula and analyzed using an unstratified log-rank test. Subgroup analysis was prespecified.

All statistical analyses were computed with the RStudio statistical software (Version 1.1.383).

## 5. Conclusions

These results suggest that the efficacy and safety of dabrafenib plus trametinib combination in patients with *BRAF* V600E-mutant metastatic NSCLC are comparable in a real-world setting and in clinical trials for both previously untreated and treated patients.

## Figures and Tables

**Table 1:** Characteristics of patients.

Characteristics	All Patients *n* = 40	1st Line *n* = 9	≥2nd Line ^a^ *n* = 31
Age (years)			
Mean (SD)	70.1 (9.8)	74.3 (9.8)	68.8 (9.6)
Range	49–94	64–94	49–84
Women, *n* (%)	22 (55)	5 (56)	17 (55)
Smoking status, *n* (%)			
Current smokers	7 (17.5)	2 (22)	5 (16)
Former smokers	20 (50)	3 (33)	17 (55)
Never-smokers	13 (32.5)	4 (44)	9 (29)
ECOG performance status at diagnosis, *n* (%)			
0–1	33 (87)	6 (67)	27 (93)
≥2	5 (13)	4 (33)	2 (7)
Missing	2	0	2
Weight loss > 5% at diagnosis, *n* (%)	29 (76)	5 (56)	24 (83)
Missing	2	0	2
Cancer-related symptoms at diagnosis, *n* (%)	36 (92)	9 (100)	27 (90)
Missing	1	0	1
Stage IV at diagnosis, *n* (%)	30 (75)	7 (78)	23 (74)
Histology, *n* (%)			
Adenocarcinoma	38 (95)	9 (100)	29 (94)
Large-cell carcinoma	1 (2.5)	0	1 (3)
Squamous cell carcinoma	1 (2.5)	0	1 (3)
PDL-1 status, *n* (%)			
PDL-1 > 1%	14 (78)	5 (83)	9 (75)
Missing	22	3	19
*BRAF* V600E at dabrafenib plus trametinib initiation, *n* (%)	40 (100)	9 (100)	31 (100)
Concomitant mutation, *n* (%)	0	0	0
Stage IV at dabrafenib plus trametinib initiation, *n* (%)	38 (95)	8 (89)	30 (97)
Number of metastatic sites, *n* (%)			
0	2 (5)	1 (12.5)	1 (3)
1	9 (23)	1 (12.5)	8 (26)
≥2	28 (72)	6 (75)	22 (71)
Missing	1	1	0
Metastatic sites, *n* (%)			
Pleura	19 (48)	4 (44)	15 (48)
Bone	16 (40)	3 (33)	13 (42)
Lung	13 (33)	1 (11)	12 (39)
Brain	5 (13)	0	5 (16)
Prior treatments (first line), *n* (%)			
Platinum-based doublet chemotherapy	-	-	26 (90)
Vemurafenib	-	-	1 (3)
Immunotherapy	-	-	2 (7)
Missing	-	-	2

^a^ Dabrafenib plus trametinib as second line, *n* = 15; third line, *n* = 8; fourth line and more, *n* = 8.
